# Revisiting the chaperonin T‐complex protein‐1 ring complex in human health and disease: A proteostasis modulator and beyond

**DOI:** 10.1002/ctm2.1592

**Published:** 2024-02-16

**Authors:** Chenglong Zeng, Shenqi Han, Yonglong Pan, Zhao Huang, Binhao Zhang, Bixiang Zhang

**Affiliations:** ^1^ Hepatic Surgery Center, Tongji Hospital, Tongji Medical College, Huazhong University of Science and Technology Wuhan China; ^2^ Clinical Medical Research Center of Hepatic Surgery at Hubei Province Wuhan China; ^3^ Hubei Key Laboratory of Hepato‐Pancreatic‐Biliary Diseases, Tongji Hospital, Tongji Medical College, Huazhong University of Science and Technology Wuhan China; ^4^ Key Laboratory of Organ Transplantation, Ministry of Education Wuhan China; ^5^ Key Laboratory of Organ Transplantation, National Health Commission Wuhan China; ^6^ Key Laboratory of Organ Transplantation, Chinese Academy of Medical Sciences Wuhan China

**Keywords:** cancer hallmarks, drug resistance, metastasis, neuropathies, proteostasis, signal transduction, TRiC/CCT

## Abstract

**Background:**

Disrupted protein homeostasis (proteostasis) has been demonstrated to facilitate the progression of various diseases. The cytosolic T‐complex protein‐1 ring complex (TRiC/CCT) was discovered to be a critical player in orchestrating proteostasis by folding eukaryotic proteins, guiding intracellular localisation and suppressing protein aggregation. Intensive investigations of TRiC/CCT in different fields have improved the understanding of its role and molecular mechanism in multiple physiological and pathological processes.

**Main body:**

In this review, we embark on a journey through the dynamic protein folding cycle of TRiC/CCT, unraveling the intricate mechanisms of its substrate selection, recognition, and intriguing folding and assembly processes. In addition to discussing the critical role of TRiC/CCT in maintaining proteostasis, we detail its involvement in cell cycle regulation, apoptosis, autophagy, metabolic control, adaptive immunity and signal transduction processes. Furthermore, we meticulously catalogue a compendium of TRiC‐associated diseases, such as neuropathies, cardiovascular diseases and various malignancies. Specifically, we report the roles and molecular mechanisms of TRiC/CCT in regulating cancer formation and progression. Finally, we discuss unresolved issues in TRiC/CCT research, highlighting the efforts required for translation to clinical applications, such as diagnosis and treatment.

**Conclusion:**

This review aims to provide a comprehensive view of TRiC/CCT for researchers to inspire further investigations and explorations of potential translational possibilities.

## Graphical abstract

1. The roles of cytosolic T‐complex protein‐1 ring complex (TRiC/CCT) in physiological and pathological processes are updated.

2. Except for modulating proteostasis as a chaperonin, TRiC/CCT regulates cell cycle, transcription and translation initiation, immortality, epigenetic changes, T‐cell immunity, autophagy and signal transduction.

3. TRiC/CCT dysfunction leads to various clinical disorders, and displays the potential to serve as an effective marker and target for pharmacological intervention for various diseases.

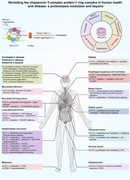

## 1 BACKGROUND

Proteostasis is regulated by a sophisticated network that influences the synthesis, folding, trafficking, disaggregation, and degradation of proteins. Within this network, the molecular chaperone system plays a central coordinating role.^1^ The molecular chaperome comprises the collective cellular folding machinery, which consists of chaperones, cochaperones and chaperonins.^2^ Most small proteins can fold spontaneously,^3^ while the majority of large proteins, which dominate the cellular proteome, rely on the molecular chaperome to adopt their native conformation. Chaperones are predominantly heat shock proteins (HSPs), such as HSP70 and HSP90, which are frequently activated under stress conditions; these proteins bind nascent proteins to promote the correct folding of these substrates and subsequently release them into the cytoplasm.^4^ Chaperonins depend on adenosine triphosphate (ATP) binding and hydrolysis to bind and fold nonnative proteins within their enclosed central chamber.^5^ Evolutionarily, chaperonins are classified into two different groups—group I and group II—based on their architecture and folding mechanism. Group I chaperonins, which include bacterial cytosolic (GroEL), mitochondrial (HSP60) and chloroplast (Cpn60) proteins, require cochaperonins (HSP10s) to facilitate protein folding. On the other hand, the group II chaperonins consist of the archaeal chaperonins called thermosomes and the eukaryotic chaperonin named T‐complex protein‐1 ring complex (TRiC/CCT).^6^ TRiC/CCT either interacts directly with substrate proteins or utilises cofactors such as prefoldin and HSP70 for substrate delivery.^7^, ^8^ TRiC/CCT is required for the folding of many essential proteins (such as the obligate folding substrates, namely, the cytoskeleton‐associated proteins tubulin^9^ and actin^10^).^5^, ^11^ In 2001, CCT subunits were first linked to liver cancer and colon cancer,^12^ Down syndrome^13^, ^14^ and Alzheimer's disease (AD).^15^ Since these discoveries, the functions of TRiC/CCT in human diseases have garnered widespread research interest.

In this review, we retrospectively examine the structure and conformation cycle mediated by TRiC/CCT. Then, we describe the versatile roles of TRiC/CCT in biological processes from the perspective of the protein interactome. Moreover, we systematically summarise TRiC‐related diseases and review the mechanisms underlying the influence of TRiC/CCT on these diseases. In particular, the potential of using TRiC/CCT as a diagnostic and prognostic marker as well as a therapeutic target is introduced, shedding light on its potential for translation from the laboratory bench to the clinical bedside.

## 2 ARCHITECTURE AND OVERALL STRUCTURE OF TRIC/CCT

**FIGURE 1:**
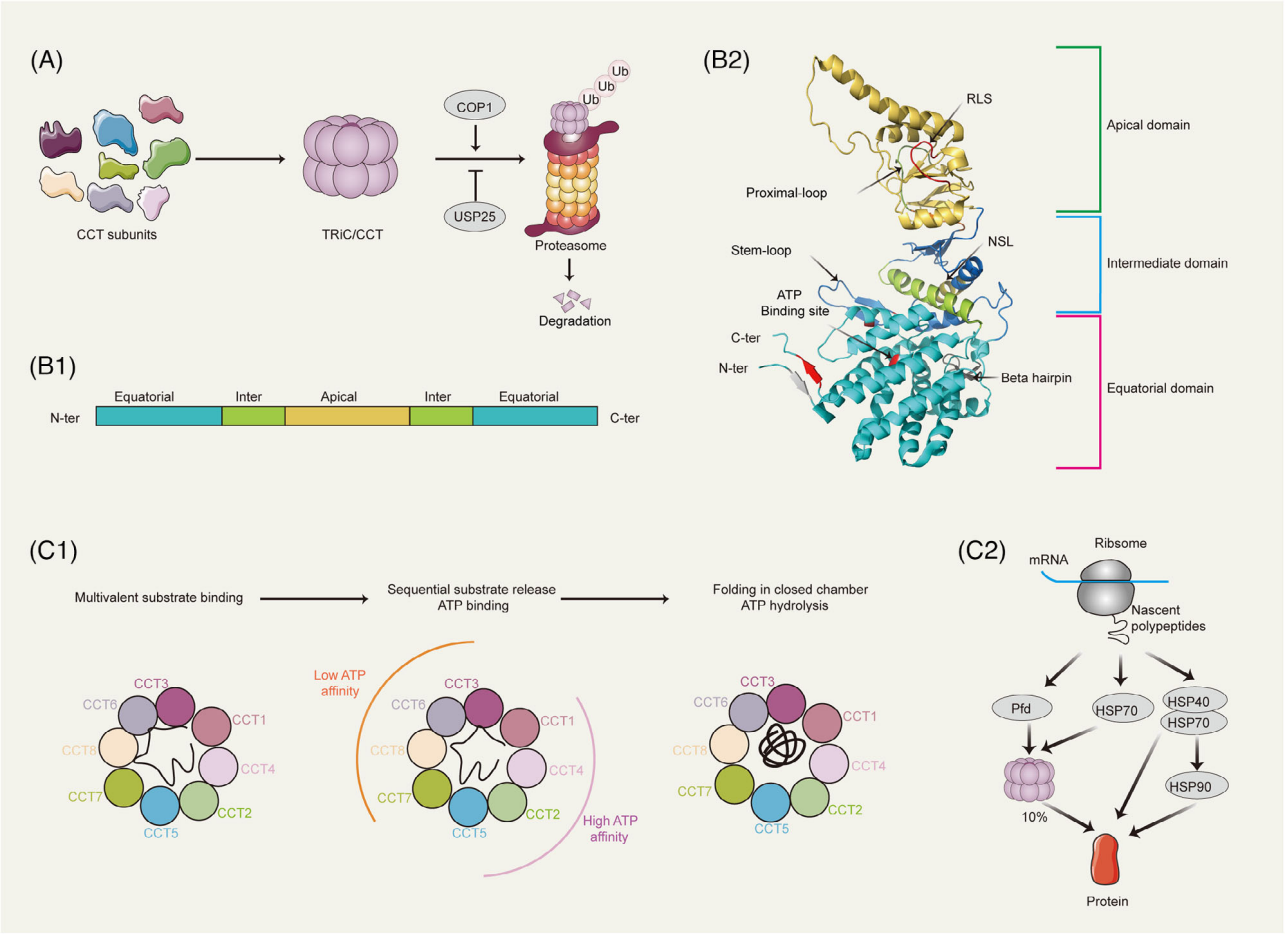
The structure and conformational cycle of the TRiC/CCT complex. (A) CCT subunits form a 1‐MDa hetero‐oligomer with eight distinct paralogous subunits, labeled CCT1–CCT8. COP1 is an E3 ubiquitin ligase that promotes TRiC/CCT degradation, while USP25, a deubiquitinating enzyme, stabilises TRiC/CCT. (B) Schematic diagrams of the secondary structure of the CCT subunits (B1). Each CCT subunit comprises three spatial domains: the apical domain, equatorial domain and intermediate domain. The ATP‐binding domain is highly conserved among the CCT subunits, whereas the substrate‐binding domain is variable. Key regions include the release loop (RSL) for substrate binding, the proximal loop (for substrate binding) and the nucleotide sensing loop (NSL) (B2). (C) The TRiC/CCT protein folding cycle. TRiC/CCT has a stable subunit arrangement (CCT 5−2−4−1−3−6−8−7) in each ring. Nonnative substrate proteins are recognised by multiple CCT subunits with varying ATP affinities. ATP binding and hydrolysis induce conformational changes, closing the folding chamber via apical domain assembly for protein folding (C1). The Hsp70–Hsp90 chaperone system for protein folding is also activated in eukaryotes (C2).

TRiC/CCT is a 1‐MDa hetero‐oligomer consisting of two stacked octameric rings that fold proteins in a manner dependent on ATP hydrolysis (Figure 1A).^16^ Phylogenetic analysis indicated that the different CCT subunit genes are the products of duplication events occurring early during the evolution of eukaryotic cells and that these products independently diverged with distinct functions.^17^, ^18^ Although the CCT subunits carry only 27%–39% identical sequences, their secondary structures are very similar. Each subunit consists of three domains: the apical domain comprising the top of the ring, which recognises a substrate and forms a lid; the equatorial domain, which contains an ATP‐binding site; and the intermediate domain, which is indispensable for ATP hydrolysis and ATP cycling^19^ (Figure 1B). The ATP‐binding domain is the most conserved domain across CCT subunits, while the substrate‐binding domain varies across subunits.^20^ Via analysis of crosslinking mass spectrometry data, TRiC/CCT was shown to have a stable subunit arrangement (CCT 2−4−1−3−6−8−7−5) in each ring, and the two rings are stacked, with a two‐fold symmetry axis centred on the inter‐ring CCT2–CCT2’ and CCT6–CCT6’ homotypic dimers.^21^

However, how CCT subunits are recruited to be in close proximity and maintain a dynamic balance for assembly has not been determined. One of the possible underlying mechanisms involves S‐palmitoylation, a posttranslational modification that sequesters CCT subunits in subcellular compartments.^22^, ^23^ S‐palmitoylation is a dynamic and reversible process. Notably, five of the eight subunits (CCT1, CCT2, CCT3, CCT4 and CCT5) can be S‐palmitoylated.^23^ This observation reminds us of the potential of S‐palmitoylation to facilitate the organised arrangement of the CCT subunits. In addition, a recent study demonstrated that TRiC/CCT assembly requires the initial assembly of the CCT subunits into a CCT5 homo‐oligomeric ring.^22^ CCT5 can form dimers with all eight subunits except for CCT8, indirectly supporting this viewpoint.^24^

The processes of TRiC/CCT disassembly and degradation are less well understood than those of its assembly. Early studies demonstrated that two octameric rings disassemble one ring at the same time to generate microcomplexes and monomers, not double rings.^25^ Analysis of the degradation of CCT subunits in mammary carcinoma cells suggested that these subunits have a half‐life of 6–8 h that is dependent on the proteasome.^26^ In addition, constitutive photomorphogenesis protein 1 (COP1),^27^ an E3 ubiquitin ligase of TRiC/CCT, and ubiquitin‐specific peptidase 25 (USP25),^28^ a deubiquitinase of TRiC/CCT, were identified (Figure 1A).

TRiC/CCT folds approximately 10% of cytosolic proteins, including many cytoskeleton‐related and crucial regulatory proteins.^29^, ^30^ TRiC/CCT is composed of eight paralogous subunits in each ring, each with distinct substrate recognition properties. This structural diversity allows TRiC/CCT to determine the topology of its bound substrate. Additionally, the CCT subunits exhibit varying affinities for ATP, with low‐affinity and high‐affinity subunits arranged separately within the ring. This spatial organisation suggests the sequential progression of the ATP‐driven conformational cycle. Furthermore, the specific arrangement of the subunits leads to noticeable charge asymmetry within the folding chamber, potentially affecting the folding sequence of the enclosed substrate. Both HSP70 and prefoldin can attach to nascent protein chains and facilitate their transfer to the chaperonin TRiC/CCT^29^, ^30^ (Figure 1C). However, the mechanism by which TRiC/CCT recognises these substrates remains unknown. TRiC/CCT substrates vary widely in both topology and molecular weight (ranging from 40^31^ to 223 kDa^32^), with few shared characteristics except for their propensity for multidomain folding, their complexity and the presence of WD‐40 repeat domains. Although the apical domains of TRiC/CCT are generally considered responsible for substrate recognition, recent studies have demonstrated that the inter‐ring septum of TRiC/CCT can bind substrates and stabilise them in the open conformation by interacting with the N‐ and C‐termini of the CCT subunits, which belong to the equatorial domains.^33^, ^34^ These observations suggest that multiple regions of the CCT subunits work together rather than individually in separate apical domains to accomplish substrate protein recognition and folding.

In general, all eight CCT subunits are required for the proper function of TRiC/CCT, and knocking down any of these subunits impairs the chaperonin activity of the complex. Knocking down a single CCT subunit can suppress the synthesis of tubulins and other CCT subunits, thus decreasing the levels of assembled protein oligomers.^35^, ^36^, ^37^ Overexpression of a single CCT subunit (CCT1 or CCT7) did not affect the overall level of assembled TRiC/CCT in wild‐type yeast cells.^38^ However, in human pluripotent stem cells, overexpressing the single subunit CCT8 enhanced the assembly of the TRiC/CCT complex.^39^ This discrepancy may be attributed to the distinct roles played by the different CCT subunits in the sequential assembly of TRiC/CCT^40^ as well as to variations in cellular backgrounds.

## 3 PROTEIN INTERACTOME AND BIOLOGICAL FUNCTIONS OF TRIC/CCT

**FIGURE 2:**
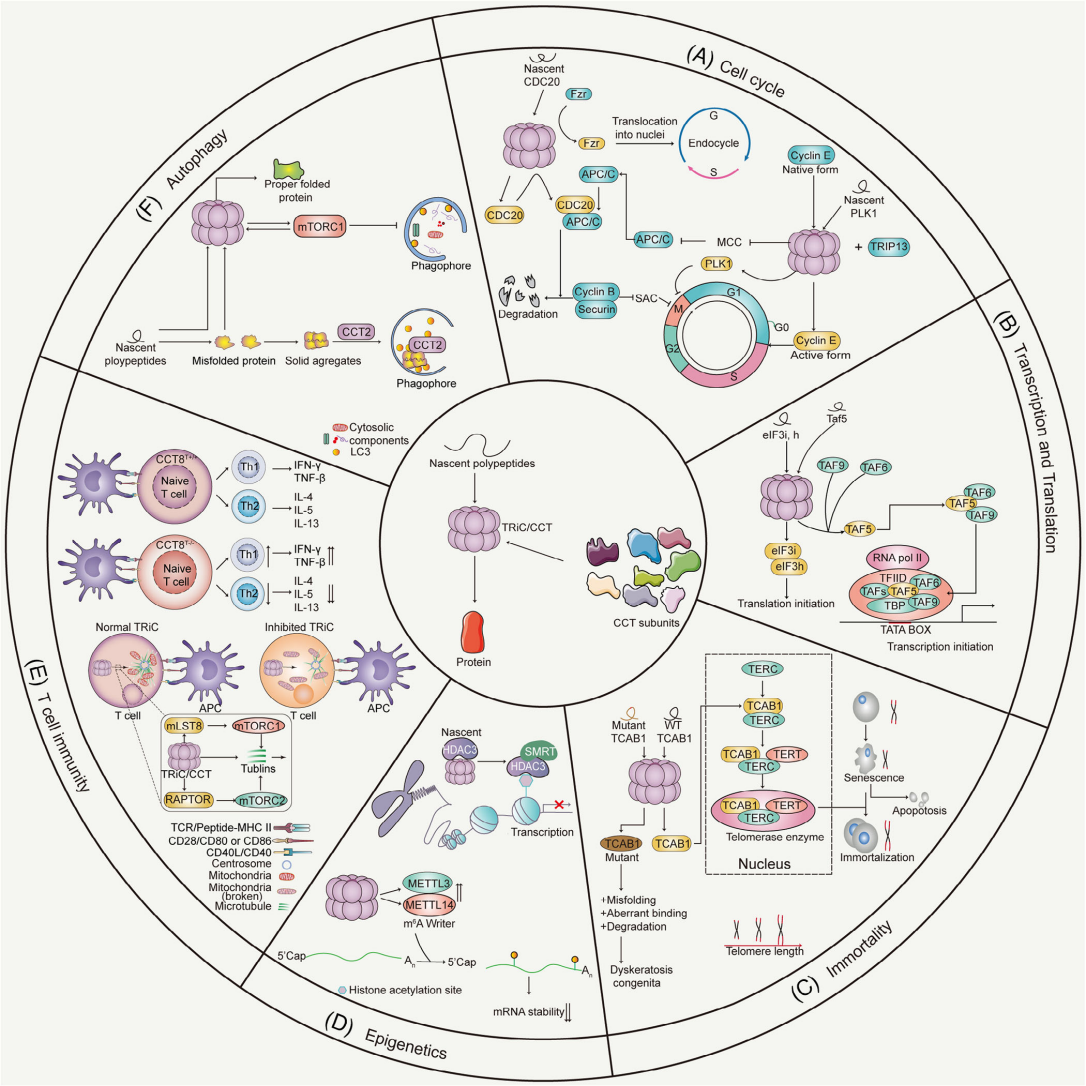
Protein interactome and biological functions of TRiC/CCT. The multifaceted functions of TRiC/CCT extend across diverse biological processes, making it a critical player in maintaining proteostasis and cellular functionality. (A) Cell cycle‐related proteins: CDC20 is a TRiC/CCT substrate. TRiC/CCT thus facilitates the assembly of the APC/C^Cdc20^ complex, thus promoting the degradation of cyclin B1 and securin and facilitating CDC20‐dependent cell cycle events. TRiC/CCT regulates the folding of Fzr, impacting endocycle transitions. CCT mediates the maturation of cyclin E, which positively controls the G1/S transition. PLK1, a cell cycle kinase, requires TRiC/CCT for proper folding. CDC20 assembles with MAD2, BUBR1 and BUB3 to form a mitotic checkpoint complex (MCC), which inhibits APC/C substrate recruitment. TRiC/CCT facilitates the disassembly of MCC, deactivating the mitotic checkpoint. (B) Transcription and translation initiation‐related proteins: TRiC/CCT transfers nascent TAF5 to TAF6–TAF9 for holo‐TFIID assembly, initiating transcription. It also aids in the correct folding and translational activity of translation initiation factor eIF3 subunits i and h. (C) Immortality‐related proteins: TRiC/CCT folds the telomerase cofactor TCAB1, which is essential for telomerase function. (D) Epigenome‐related proteins: TRiC/CCT is required for the activation of HDAC3, which, in turn, inhibits gene transcription. TRiC/CCT thus participates in mRNA modification by mediating m^6^A modification and regulating METTL3 and METTL14 protein levels. (E) T‐cell immunity‐related proteins: TRiC/CCT influences the T‐cell phenotype and cytokine production, impacting T‐cell activation and function. (F) Autophagy‐related proteins: TRiC/CCT plays a role in autophagy regulation, including mTORC1 complex activation, and serves as an autophagy receptor for misfolded proteins.

As a multifunctional protein, TRiC/CCT associates with numerous proteins to execute diverse functions. TRiC/CCT plays a critical role in regulating the cell cycle, transcription and translation initiation, cellular immortality, epigenetic changes, T‐cell immunity, autophagy and signal transduction (Figure 2).

### 3.1 Cell cycle‐related proteins

TRiC/CCT is involved in cell cycle regulation, specifically in the G1/S transition and in the mitotic (or spindle assembly) checkpoint system. TRiC/CCT regulates the cell cycle through two mechanisms: one involving substrate folding, and the other relying on protein interactions. Certain cell cycle‐related proteins, such as Polo‐like kinase 1 (PLK1), cell division cycle 20 homolog (CDC20), cyclin E and Fzr, are substrates of TRiC/CCT and are folded by this complex. PLK1, a kinase involved in mitosis, was identified as the TRiC/CCT substrate that mediates correct folding. Knocking down CCT1 reduced PLK1 expression, leading to G2/M arrest.^41^ CDC20 functions as the critical factor that controls the spindle assembly checkpoint to maintain genome stability, ensuring the correct separation of chromosomes during mitosis.^42^ TRiC/CCT is indispensable for the proper folding of CDC20 and thus for CDC20‐dependent cell cycle events, such as the separation of sister chromatids and checkpoint exit during mitosis. Additionally, CCT2 and CCT5 are essential for the stabilisation of CDC20 to mediate cell cycle arrest.^43^, ^44^, ^45^ However, TRiC/CCT is crucial for the maturation of cyclin E, which positively controls the G1/S transition.^46^ Endocyclic cells and tumour cells proliferate via a unique cell cycle mechanism called mitotic‐to‐endocycle switching (MES).^47^, ^48^ The TRiC/CCT supports MES by regulating Fzr folding.^49^ In addition, CCT5 interacts with cyclin D1, which, as a cell cycle‐related protein, crucially accelerates the G1/S transition, and overexpression of cyclin D1 increases CCT5 expression.^50^ Moreover, CCT interacts with the ATPase thyroid hormone receptor interactor 13 (TRIP13)^51^ and with cyclin D1^50^ to regulate the cell cycle. TRiC/CCT interacts with TRIP13 to facilitate the complete disassembly of mitotic checkpoint complexes, thus deactivating mitotic checkpoints^51^ (Figure 2A).

### 3.2 Transcription and translation initiation‐related proteins

The general transcription factor IID complex (TFIID) is a cornerstone of eukaryotic gene regulation for transcription initiation.^52^ TATA‐box‐binding protein‐associated factor 5 (TAF5) is the presumed central scaffold within TFIID.^53^ TRiC/CCT folds nascent TAF5 and releases prefolded TAF5 via TAF6–TAF9 binding, resulting in the formation of a distinct TAF5–TAF6–TAF9 complex. This complex binds additional TAFs and TATA‐binding protein (TBP) to assemble the functional holo‐TFIID complex, thereby initiating transcription (Figure 2B).^54^ Interestingly, TRiC/CCT associates with multiple viral RNA polymerases, such as hepatitis C virus NS5B,^55^ and influenza A virus PB1 and PB2.^56^, ^57^ Eukaryotic RNA polymerase subunits (POLR3A/POLR1C) have also been identified through interactome analysis as potential binding partners of TRiC/CCT.^58^ In addition, CCT mediates the correct folding and translation activity of two subunits of the translation initiation factors eIF3i and eIF3h. TRiC/CCT enhances internal ribosome entry segment‐dependent translation initiation through eIF3h, and it increases both internal ribosome entry segment‐ and cap‐dependent translation initiation via eIF3i^59^ (Figure 2B).

### 3.3 Immortality‐related proteins

Proteostasis is a common characteristic that is linked to organismal longevity and the immortality of pluripotent stem cells. Accelerated assembly of TRiC/CCT has been identified as an intrinsic characteristic of pluripotent stem cells. Additionally, CCT8 overexpression in somatic tissues was found to extend the lifespan of *Caenorhabditis elegans* in a TRiC/CCT‐dependent manner.^39^ Telomerase can accomplish the immortalisation of a variety of cells.^60^ The active telomerase enzyme is composed of a catalytic core (the telomerase RNA component, TERC and telomerase reverse transcriptase) and a small number of additional proteins.^61^, ^62^ Importantly, TRiC/CCT has been identified as an essential regulator of the trafficking of telomerase. TRiC/CCT mediates the folding of telomerase Cajal body protein 1 (TCAB1), thereby mediating the trafficking of TERC and small Cajal body RNAs. TRiC/CCT deficiency results in loss of the TCAB1 protein, mislocalisation of telomerase and small Cajal body‐specific RNAs (scaRNAs) to nucleoli, and failure of telomere elongation.^63^ Moreover, in patients with dyskeratosis congenita, mutations in TCAB1 impair its folding via TRiC. Mutation of TCAB1 markedly increases its association with TRiC/CCT, but significantly reduces its binding to TERC, disrupting the function of telomerase and causing severe disease^63^ (Figure 2C).

### 3.4 Epigenetic modification‐related proteins

Several studies have shown that TRiC/CCT regulates gene transcription via epigenetic alterations—histone acetylation and N6‐adenosine methylation (m^6^A). Histone deacetylase 3 (HDAC3) reverses the effects of histone acetyltransferases via the deacetylation of histones, and thus represses gene transcription. The enzymatic activity of HDAC3 requires the presence of silencing mediator of retinoid and thyroid hormone receptor (SMRT).^64^ For activation of its enzymatic activity, HDAC3 must first bind to TRiC/CCT, after which TRiC/CCT is displaced by SMRT.^65^ Dysfunction of TRiC/CCT can impair the enzymatic activity of HDAC3, thereby dysregulating gene transcription. Furthermore, TRiC/CCT mediates mTORC1‐dependent m^6^A methylation to impede autophagy.^66^ Depleting only TRiC/CCT was found to reduce the METTL3 and METTL14 protein levels, increase the stability of transcripts of autophagy‐related genes and promote autophagy (Figure 2D).

### 3.5 T‐cell immunity‐related proteins

Mammalian T lymphocytes are activated by antigen‐presenting cells, which are involved in the formation of immunological synapses (ISs), polarised structures supporting cell–cell communication.^67^ Full activation of T lymphocytes largely depends on centrosome polarisation.^68^ Recent studies indicate that TRiC/CCT participates in adaptive immunity by regulating T‐cell activation and polarity. For example, TRiC/CCT influences the metabolic status of activated T cells by facilitating protein synthesis and folding, directly affecting the ability of tubulin to assemble into microtubules (Figure 2E).^69^ Similarly, T‐cell asymmetry during IS contact in mammals requires TRiC/CCT and mammalian target of rapamycin (mTOR). The IS governs cytoskeletal dynamics while also regulating mitochondrial fate, respiration and metabolic rates. Ultimately, communication at the IS leads to cellular reprogramming events that are linked to the functional outcomes of CD4^+^ T cells.^70^ In human CD4^+^ T cells, transient knockdown of TRiC/CCT reduces calcium flux and the activation of nuclear factor of activated T cells (NFAT). TRiC/CCT is involved in regulating the internalisation of Orai1, the principal channel for store‐operated calcium entry, through protein‒protein interactions at the plasma membrane during IS formation in CD4^+^ T cells. Therefore, TRiC/CCT increases calcium flux and NFAT activation. Through this mechanism, CCT governs both T‐cell activation and polarity.^71^ Additionally, deficient Th2 cell polarisation was observed in CCT8^T−/−^ mice (designated CD4^Cre^::CCT8^fl/fl^ mice). This protection was impaired after the expulsion of *Heligmosomoides polygyrus* after a brief primary infection followed by re‐exposure to parasite larvae. T cells that lacked CCT8 acquired a Th1‐cell phenotype and secreted IFN‐γ after activation, suppressing the type 2 immune response and decreasing the cell proliferation rate, which ultimately compromised protective immunity against *H. polygyrus* ^72^ (Figure 2E). These findings demonstrate that normal T‐cell biology relies on TRiC/CCT and that dysfunction of TRiC/CCT impairs adaptive immunity.

### 3.6 Autophagy‐related proteins

Autophagy is an evolutionarily conserved, tightly regulated, and lysosome‐dependent catabolic process in eukaryotes that degrades unnecessary or dysfunctional cellular components and recycles metabolic substrates.^73^ The mTORC1 complex can regulate autophagy. An early study indicated that mTORC1 activates TRiC/CCT.^74^ In turn, TRiC/CCT regulates mTORC1 by folding mammalian lethal with sec thirteen 8 (mLST8).^33^, ^75^ Thus, TRiC/CCT plays complex roles in autophagy under different conditions, but its impacts on health and disease remain unclear. Tang et al. discovered that TRiC/CCT modulates m^6^A modification to inhibit autophagy.^66^ In contrast, CCT2 was identified as a novel autophagy receptor that promotes the autophagic degradation of protein aggregates (especially solid aggregates)^76^ (Figure 2F).

### 3.7 Signalling pathway protein‐related proteins

G protein‐coupled receptors (GPCRs), among the most influential cell‐surface proteins, mediate cellular responses to extracellular signals such as hormones, neurotransmitters, sensory cues and drug signals, contributing to nearly all aspects of cell physiology.^77^ GPCRs activate G proteins, which are composed of α, β and γ subunits, in turn transducing signals to downstream effectors. In 2002, TRiC/CCT was identified as the third binding partner of Phosducin‐like protein (PhLP), which regulates the activity of G protein beta‐gamma (Gβγ) subunits. PhLP inhibits the folding activity of TRiC/CCT by directly competing with it for binding to protein substrates, such as beta‐actin.^78^ Manipulating the level of PhLP may be a novel strategy for controlling TRiC‐dependent protein folding. In 2006, Wells et al. reported that TRiC/CCT is indispensable for the assembly of Gβγ subunits.^79^ Additionally, Plimpton et al. confirmed that TRiC/CCT facilitates the assembly of Gβγ subunits, as determined via cryo‐electron microscopy; TRiC/CCT cooperates with the cochaperone PhLP, which mediates the release of Gβ from TRiC/CCT.^80^ This finding suggests that the different chaperone classes and their cofactors cooperate to fold proteins, thereby maintaining proteostasis. Furthermore, CCT7 interacts with two GPCRs—the β2‐adrenergic receptor and the β‐isoform of the thromboxane A2 receptor. CCT7 deletion reduces the cell surface expression of these receptors and alters their intracellular distribution.^81^ Thus, TRiC/CCT plays a role in affecting intercellular communication. Moreover, TRiC/CCT interacts with numerous other proteins, including mothers against decapentaplegic homolog 2 (SMAD2), β‐catenin, mLST8 and yes‐associated protein 1 (YAP), and is either an upstream molecule or downstream target in various classical signalling pathways, such as the transforming growth factor (TGF)‐β,^82^ Wnt/β‐catenin,^83^, ^84^ mTOR^33^, ^74^, ^75^, ^85^, ^86^ and Hippo‐YAP^87^ signalling pathways. The contribution of TRiC/CCT to signal transduction related to disease pathology is detailed in the following section.

## 4 TRIC/CCT IN DISEASES

Increasing evidence indicates that TRiC/CCT is closely related to tumours and multiple nonmalignant diseases, such as neuropathies and cardiovascular diseases.

### 4.1 TRiC/CCT and neuropathies

**FIGURE 3:**
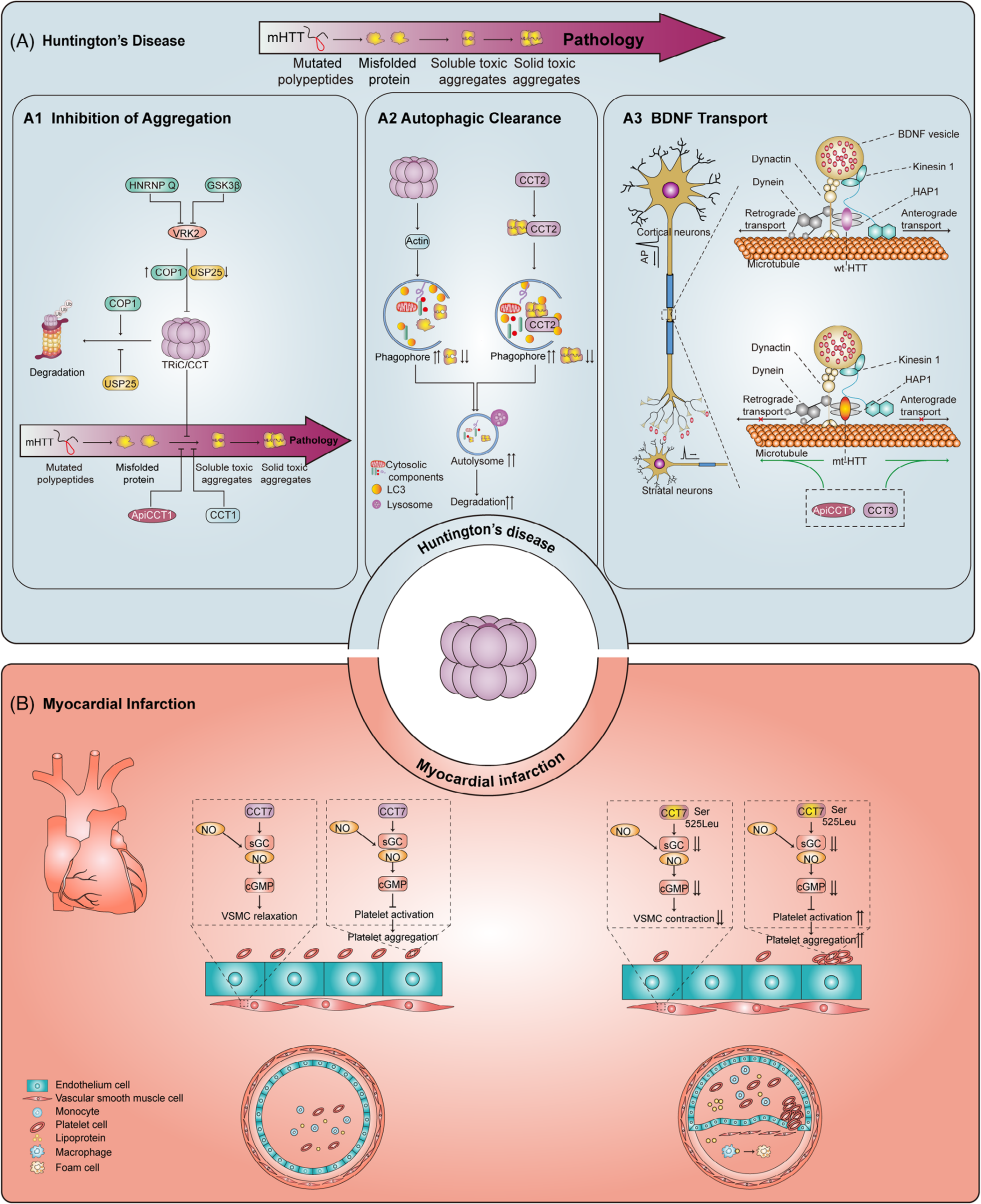
Molecular mechanisms underlying the functions of TRiC/CCT in Huntington's disease and myocardial infarction. (A) TRiC/CCT in Huntington's disease: TRiC/CCT plays a role in the inhibition of aggregation in Huntington's disease; monomeric mutant huntingtin (mHtt) folds incorrectly and aggregates, but TRiC/CCT facilitates proper mHtt folding and suppresses its aggregation, inhibiting disease progression (A1). TRiC/CCT also plays a role in autophagic clearance. Autophagy, regulated by TRiC/CCT, also attenuates mHtt aggregation. Additionally, CCT2 acts as a novel autophagy receptor, promoting the degradation of mHtt aggregates (A2). TRiC/CCT plays a role in BDNF transport. Deficits in BDNF transport can lead to striatal atrophy in patients with Huntington's disease. Wild‐type huntingtin regulates BDNF vesicle transport, while mutant huntingtin disrupts this process. However, CCT3 and ApiCCT1 increase the BDNF axonal transport rate, alleviating striatal atrophy (A3). (B) TRiC/CCT in myocardial infarction: CCT7 stabilises soluble guanylyl cyclase (sGC), regulating the NO/sGC/cGMP pathway, inducing vasodilation and inhibiting platelet aggregation. A heterozygous mutation in CCT7 (Ser525Leu) reduces cGMP stability, weakening the effect of sGC‐dependent NO signalling in vascular smooth muscle and platelets, leading to thrombus formation and causing myocardial infarction.

Neuropathies are complicated diseases that share certain pathogenetic processes. One of these processes involves the failure of the proteostasis network, leading to aberrant accumulation of cytotoxic protein aggregates in neurons.^88^ After cell division, the concentrations of misfolded proteins in neurons cannot be decreased, because these neurons are postmitotic. TRiC/CCT is critical for neuronal activity, and dysfunction of TRiC/CCT is evident in multiple neuropathies, including Huntington's disease (HD), Parkinson's disease and AD (Figure 3A).

#### 4.1.1 Huntington's disease

HD is a progressive neurodegenerative disorder characterised by movement difficulties, psychiatric symptoms and cognitive decline caused by abnormal accumulation of pathogenic proteins (the polyglutamine [polyQ]‐extended mutant huntingtin protein [mHTT]).^89^ TRiC/CCT can exert a beneficial effect on HD by preventing expanded polyQ aggregation and the resulting induced toxicity in neuronal cells, reinforcing autophagic clearance to restrict neuropathogenic protein aggregation, and increasing brain‐derived neurotrophic factor (BDNF) expression to ameliorate striatal atrophy. For instance, Behrends et al. and Kitamura et al. discovered that TRiC/CCT decreased amyloid fibre assembly and attenuated the toxicity of the polyQ‐extended mHTT that causes HD.^90^, ^91^ Thereafter, Shahmoradian et al. provided the structural basis for these studies, finding that TRiC/CCT caps mHTT (Q51) fibril tips through the apical domains of CCT subunits and encapsulates mHTT oligomers within its chamber.^92^ In contrast, promoting the degradation of TRiC/CCT can lead to HD. Vaccinia‐related kinase 2 (VRK2) mediates the degradation of TRiC/CCT to induce mHTT aggregation by recruiting the E3 ligase COP1 and inhibiting USP25.^27^, ^28^ By inhibiting VRK2, glycogen synthase kinase 3β (GSK3β) suppresses mHTT aggregation.^93^ Heterogeneous nuclear ribonucleoprotein Q (HNRNP Q) decreases the mRNA stability of VRK2. In response to a reduction in the HNRNP Q level, the CCT4 protein level markedly decreases, which suppresses the function of TRiC/CCT, resulting in increased polyQ aggregation in human and mouse neurons.^94^ These studies indicated that TRiC/CCT dysfunction causes HD and that VRK2 may be a key regulatory protein in this pathological process. In addition, overexpression of CCT1 attenuates mHTT aggregation and alleviates mHTT‐induced toxicity in neuronal cells. The apical domain of CCT1 (ApiCCT1) reduces mHTT aggregation independently of TRiC/CCT in vitro.^38^ In particular, Sontag's group^95^ demonstrated that recombinant ApiCCT1 (ApiCCT1(r)) protein may be a promising drug for attenuating the acquisition of the cell phenotypes induced by mHTT. Moreover, the chaperonin‐like CCT5 complex inhibits mHTT protein aggregation.^96^ These studies indicate that some CCT subunits may mediate the functions of TRiC/CCT and have evolved binding affinities for substrates. CCT mimetics have shown potential therapeutic value for patients with HD. Previously, researchers assumed that TRiC/CCT regulated mHTT aggregation via direct binding and modulation of its folding function. However, insufficient clearance via autophagy may also account for mHTT aggregation. TRiC/CCT inhibits neuropathogenic protein aggregation via autophagy‐based clearance by regulating the actin cytoskeleton and lysosome biogenesis.^37^ The CCT subunits also inhibit mHTT protein aggregation through autophagy. In HD pathogenesis, the toxicity of mHTT aggregates exceeds the capacity of cells to maintain proteostasis, ultimately resulting in the formation of irreversible solid aggregates or pathological deposits. However, established aggrephagy receptors exhibit a preference for liquid‐like aggregates over solid‐like aggregates. Interestingly, Ma et al. discovered that CCT2 is a novel autophagy receptor that specifically promotes the autophagic degradation of solid protein aggregates independent of TRiC/CCT.^76^ In the context of neurodegenerative diseases, CCT2 may exert dual beneficial effects by functioning as a component of TRiC/CCT to prevent protein aggregation and as a novel autophagy receptor to clear solid protein aggregates. In addition, corticostriatal atrophy, in which neurons in corticostriatal circuits are lost, is a manifestation of HD. Deficits in BDNF in the corticostriatal circuit may lead to striatal atrophy. Interestingly, both CCT3 and ApiCCT1 were found to increase BDNF axonal transport and rescue striatal atrophy in the context of HD.^97^ Regarding the potential role of TRiC/CCT in axonal transport in HD, CCT5 was found to increase axonal transport function by inducing a pause to allow retrograde transport of BDNF in wild‐type neurons.^98^ The above findings support the idea that TRiC/CCT or CCT subunits may exert beneficial effects by decreasing the level of the toxic mHTT protein and increasing BDNF axonal transport in patients with HD (Figure 3A). Recently, clinical trials and approved therapies for HD have focused on the relief of symptoms. Treatments directed at inhibiting the accumulation of toxic products of the mutant HTT gene may constitute a direct strategy for targeting the root cause of HD pathogenesis.^99^ Targeting CCT to clear mHTT may be a promising therapeutic strategy specific for HD to prevent off‐target effects and alleviate side effects.

#### 4.1.2 Parkinson's disease

Targeting TRiC/CCT may also be an interesting therapeutic strategy for Parkinson's disease. As reported in 2015, CCT2 was upregulated after treatment with 1‐methyl‐4‐phenylpyridinium in a Parkinson's disease cell line.^100^ TRiC/CCT inhibited the assembly of amyloid fibres of α‐synuclein A53T, a mutant protein that causes Parkinson's disease, thereby reducing α‐syn A53T oligomer‐mediated cytotoxicity.^101^

#### 4.1.3 Alzheimer's disease

AD is the leading cause of cognitive impairment in the elderly population. The involvement of TRiC in Alzheimer's disease was identified through a patient genome‐wide association study and confirmed by RNA interference screening of Aβ‐expressing *C. elegans*. Additionally, CCT1 and CCT8 were identified as suppressors of the Aβ‐paralysis phenotype in an RNA interference library screen.^102^ Recently, CCT5 was identified as a protein that is specifically carbonylated in the early stages of AD. The oxidation level of CCT5 was significantly increased in AD mice compared to control mice. Therefore, the oxidation of CCT5, which results in loss of TRiC function, may lead to improper protein folding and subsequent protein aggregation.^103^ Moreover, through a microarray analysis to identify differentially expressed genes in patients with Alzheimer's disease, CCT2 was identified as a potential biomarker with diagnostic value.^104^

### 4.2 TRiC/CCT and cardiovascular diseases

Myocardial infarction (MI) is a leading cause of death worldwide and is frequently caused by the rupture of atherosclerotic plaques.^105^ The damaged heart undergoes pathological remodelling, resulting in a decline in contractile function and, typically, heart failure.^106^ A CCT7 heterozygous mutation was discovered in extended members of a family with a history of MI. Mechanistically, CCT7 stabilises soluble guanylyl cyclase, and CCT7 mutation affects the stability of cyclic guanosine monophosphate, thereby weakening the effect of soluble guanylyl cyclase‐dependent nitric oxide signalling to promote thrombus formation and cause MI^107^ (Figure 3B). Reversing this defect may help to reduce MI risk. In addition, lectin‐like oxidised low‐density lipoprotein receptor‐1 (LOX‐1) is a scavenger receptor that plays a critical role in atherosclerosis development by binding and degrading oxidised low‐density lipoprotein (OxLDL).^108^ CCT1 and CCT4 bind to the LOX‐1 cytoplasmic domain; however, OxLDL was found to suppress this interaction in human umbilical vein endothelial cells.^109^

## 5 CCT SUBUNITS IN CANCERS

Evidence indicates that rapidly proliferating tumour cells exhibit increased requirements for protein folding and trafficking to maintain their basic physiology.^1^ CCT subunits are more highly expressed (by approximately two‐fold) in cancer cell lines than in normal cells, as determined via analysis of tumour cell lines and normal cell lines from different organs.^110^ CCT1‐8 was also found to be overexpressed in multiple types of tumours (Table 1). Each subunit of CCT may play multiple important roles in tumours. First, CCT subunits form the TRiC/CCT complex, facilitating normal folding of proteins and preventing abnormal protein aggregation, thereby promoting proteostasis.^1^ Second, they regulate the assembly and disassembly of TRiC/CCT and influence its protein folding activity.^75^, ^111^, ^112^ Finally, they modulate signalling pathways to promote tumour occurrence and development.^82^, ^113^, ^114^ Here, we describe these functions as they relate to the hallmarks of cancer.

**TABLE 1:** Clinical relevance of CCT subunits.

Subunit	Tumour type	Mechanism	Clinical risk	Ref
CCT1	HCC	Wnt7b/β‐catenin pathway, through P53, promotes proliferation and metastasis	High CCT1 expression predicts a shorter OS time	^84^
CCT1/2	HCC and CRC	–	–	^12^
CCT1	BC	FGFR2‐PI3K‐AKT‐CCT1 pathway, promotes proliferation	High CCT1 expression predicts a shorter OS time	^154^
CCT2	BC	Target of the cytotoxic activity of CT20p	High CCT2 expression predicts a shorter OS time	^155^
	BC	–	High CCT2 expression predicts a shorter OS time	^156^
	BC	AKT–GSK3β–β‐catenin and XIAP‐Survivin pathways, facilitates chemoresistance and metastasis	–	^83^
	CRC	Hedgehog signalling pathway, through Gli‐1, promotes proliferation and metastasis	High CCT2 expression predicts a shorter OS time	^136^
	LUAD	Targeting the β‐tubulin/CCT‐β complex causes apoptosis and inhibits invasion/migration	–	^128^
	GBC	–	High CCT2 expression predicts a shorter OS time	^157^
	TNBC, CRC, GC	Targeting the β‐tubulin/CCT‐β complex induces apoptosis through the MAPK pathway	–	^127^
CCT2/3	CRC	–	High CCT2/3 expression indicates a higher Dukes’ stage and a shorter OS time	^158^
CCT3	HCC	Promotes proliferation	High CCT3 expression is indicative of a larger tumour size, poorer pathological differentiation, an advanced TNM stage and a shorter OS time	^119^
	HCC	Hippo pathway, through YAP and TFCP2, promotes proliferation	High CCT3 expression indicates a shorter OS time	^87^
	HCC	IL6/STAT3 signalling pathway, promotes proliferation	High CCT3 expression indicates a shorter OS time	^159^
	BC	NF‐kB pathway, promotes proliferation and migration	–	^160^
	BC	Wnt/β‐catenin pathway, though miR‐223, promotes proliferation	High CCT3 expression is indicative of a higher TNM stage and a shorter OS time, and is correlated with the progesterone receptor expression level	^161^
	NSCLC	Hippo pathway, through YAP1, promotes proliferation and migration	High CCT3 expression indicates a shorter OS time	^162^
	LUAD	AKT pathway, slc7a11‐mediated ferroptosis suppression, promotes proliferation	High CCT3 expression indicates shorter OS and DFS times	^163^
	LUAD	Glycolysis and EIF3G‐mediated cytoplasmic translation, promotes proliferation	High CCT3 expression indicates a shorter OS time	^150^
	LUAD	JAK2/STAT3 pathway, facilitates chemoresistance	High CCT3 expression indicates a shorter DFS time	^143^
	CCA	–	–	^164^
	GC	Promotes proliferation	–	^123^
	Cervical cancer	FN1, promotes proliferation and migration	High CCT3 expression indicates a shorter DFS time	^165^
	HNSCC	–	High CCT3 expression indicates a shorter OS time	^166^
	Melanoma	CDK1, promotes proliferation	High CCT3 expression indicates a higher clinical stage	^167^
	Multiple myeloma	–	High CCT3 expression predicts shorter event‐free survival and OS times	^168^
	Breast and prostate cancers	Inhibits apoptosis	–	^169^
CCT4	HCC	Cdc20, promotes proliferation	–	^170^
	ESCC	AMPK/AKT/NRF2 pathway, facilitates glucose metabolism and chemoresistance	High CCT4 expression indicates a higher pathological grade and a shorter OS time	^114^
	Osteosarcoma	STAT3, inhibits apoptosis	High CCT4 expression indicates a shorter OS time	^111^
CCT5	HCC	CDK2, 4 and 6 as well as cyclinA2, B1, D1, D3 and E2, promotes proliferation	High CCT5 expression indicates a higher clinical stage and a shorter OS time	^117^
	GC	Wnt/β‐catenin pathway, promotes proliferation and lymph node metastasis	High CCT5 expression predicts more lymph node metastasis, a higher TNM stage, and shorter median survival and recurrence‐free survival times	^133^
	LUAD	PI3K/AKT pathway, promotes migration	High CCT5 expression indicates a higher TNM stage	^50^
CCT6A	HCC	–	High CCT6A expression predicts a higher BCLC stage, a shorter OS time and an elevated AFP level	^171^
	HCC	Cyclin D, promotes proliferation	High CCT6A expression indicates shorter OS and DFS times	^116^
	CRC	Promotes proliferation and migration	High CCT6A expression indicates a shorter OS time	^172^
	NSCLC	TGFβ/SMAD2 pathway, promotes metastasis	–	^82^
	NSCLC	–	High CCT6A expression predicts more lymph node metastasis, a higher TNM stage, and shorter OS and DFS times	^173^
	GC	–	High CCT6A expression indicates a higher TNM stage and a shorter OS time	^174^
	Astrocytoma	–	High CCT6A expression indicates a higher WHO grade and a shorter OS time	^175^
	Cervical cancer	–	High CCT6A expression is indicative of more lymph node metastasis, a higher FIGO stage and a shorter DFS time	^176^
	Melanoma	Facilitates chemoresistance	–	^177^
CCT8	HCC	GRP94/CCT8/c‐Jun pathway, promotes migration	–	^178^
	HCC	CDK2 and cyclin E, promotes proliferation	High CCT8 expression is associated with a larger tumour size, poorer histologic grade and a shorter OS time	^118^
	CRC	p53 pathway, promotes proliferation and metastasis	High CCT8 expression is associated with more lymph node metastasis, a higher clinical stage and a shorter OS time	^113^
	Glioma	Promotes proliferation and migration	High CCT8 expression indicates a higher WHO grade and a shorter OS time	^139^
	ESCC	α‐Actin and β‐tubulin, promotes migration	High CCT8 expression predicts a deeper tumour depth, more lymph node metastasis, poorer pathological grade and a shorter OS time	^140^

Abbreviations: AFP, alpha‐fetoprotein; BC, breast cancer; CCA, cholangiocarcinoma; CRC, colorectal cancer; DFS, disease‐free survival; ESCC, oesophageal squamous cell carcinoma; GBC, gallbladder cancer; GC, gastric cancer; HCC, hepatocellular carcinoma; HNSCC, head and neck squamous‐cell carcinoma; LUAD, lung adenocarcinoma; NSCLC, non‐small cell lung cancer; OS, overall survival; TNBC, triple‐negative breast cancer.

### 5.1 Cell cycle

**FIGURE 4:**
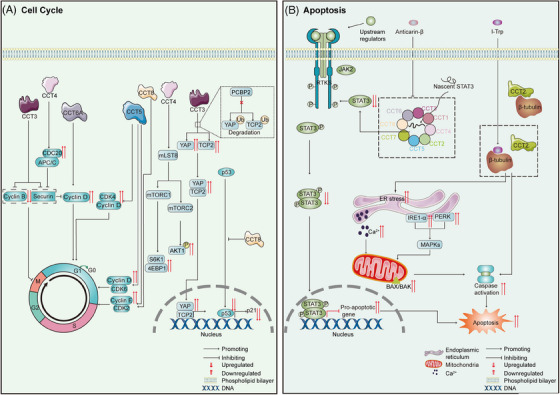
TRiC/CCT in the cell cycle and apoptosis. (A) TRiC/CCT in cell cycle control. From left to right: In hepatocellular carcinoma, CCT3 increases the degradation rate of securin and cyclin B1, thereby regulating the G2/M transition. CCT4 activates APC^Cdc20^, accelerating securin degradation and the G2/M transition. CCT6A regulates cyclin D, promoting the G1/S transition and cell proliferation. CCT5 promotes cell proliferation by positively regulating cyclin A2, B1, D1, D3 and E2 and CDK2, 4 and 6. CCT8 increases cyclin E and CDK2 expression, promoting cell proliferation. In glioblastoma cells, CCT4 regulates mLST8 to modulate the mTOR pathway. In hepatocellular carcinoma cells, CCT3 inhibits PCBP2, stabilising YAP and TCP2. Moreover, CCT8 promotes colorectal cancer cell proliferation by preventing wild‐type p53 nuclear translocation. (B) TRiC/CCT in apoptosis. From left to right: Anticarin‐β induces apoptosis in osteosarcoma cells by inhibiting CCT4 and decreasing STAT3‐mediated antiapoptotic effects. I‐Trp disrupts the β‐tubulin‐CCT2 interaction, inducing apoptosis via caspase activation in highly metastatic non‐small cell lung cancer cells. Moreover, I‐Trp activates the unfolded protein response (UPR) in various cancer cells. During the UPR, two of the three major endoplasmic reticulum (ER) stress sensors, PERK and IRE1‐α, undergo upregulation. Subsequently, they activate MAPK family proteins (MAPKs). Concurrently, the intracellular Ca^2+^ level increases, leading to disruption of mitochondria. Ultimately, these events trigger caspase activation, leading to cellular apoptosis.

Cells in all living organisms undergo cycles of growth, replication and division. The cell cycle is regulated by cyclin‐dependent kinase (CDK) activity and APC/CCdc20 activity.^115^ As mentioned before, TRiC/CCT regulates cell cycle‐related protein activity, and the CCT subunits and TRiC/CCT share certain functions (Figure 4A). In hepatocellular carcinoma (HCC) cells, CCT6A knockdown downregulates cyclin D to suppress proliferation by decelerating the G1/S transition.^116^ In addition, CCT5 promotes proliferation by positively regulating multiple cell cycle regulators, including cyclin A2, cyclin B1, cyclin D1, cyclin D3 and cyclin E2, as well as CDK2, CDK4 and CDK6, in HCC cells.^117^ Similarly, the expression of proliferating cell nuclear antigen, cyclin E and CDK2, which are normally high in proliferating cells, is markedly reduced in CCT8‐knockdown HCC cells.^118^ CCT3 depletion in HCC cells inhibits cell cycle progression by attenuating the degradation of securin and cyclin B1 to delay mitotic exit.^119^ Under physiological conditions, wild‐type p53 arrests the cell cycle until DNA damage is repaired, thus preventing DNA‐defective cells from acquiring a malignant phenotype. However, cell cycle control is lost in cells with mutated p53, resulting in the transformation of nonmalignant cells into cancerous cells.^120^ Because TRiC/CCT mediates the folding of wild‐type p53,^121^ CCT subunits promote cell cycle progression by regulating the P53 signalling pathway. KEGG analysis of breast cancer cells revealed enrichment of CCT2‐related genes in the cell cycle and p53 signalling pathways.^122^ However, the exact relationship between CCT2 and p53 in breast cancer is still unknown. Interestingly, in colorectal cancer (CRC) cells, overexpression of CCT8 significantly increases cell proliferation by preventing wild‐type p53 from being transported into the nucleus, contributing to p53 inactivation.^113^ In gastric cancer cells, CCT3 knockdown inhibits cell cycle progression, an effect associated with downregulation of cyclin D3 and TAK1. However, CDK2 and CDK6 are upregulated.^123^ These findings show that many cell cycle‐related proteins are regulated by CCT subunits in cancer cells.

### 5.2 Apoptosis

Recent studies have indicated that targeting CCT subunits in tumours promotes apoptosis by regulating tubulin^112^ and signal transducer and activator of transcription 3 (STAT3) activity.^111^ Targeting microtubules with a stabiliser (paclitaxel) or a destabiliser (vinca alkaloids) is a pharmaceutically validated anticancer strategy owing to the important role of microtubules in mitosis.^124^ As tubulin is an obligate folding substrate of TRiC/CCT,^125^ a novel compound targeting β‐tubulin/CCT2, *N*‐iodoacetyl‐tryptophan (I‐Trp), was developed in 2009. I‐Trp induces apoptosis by activating caspase‐3 and caspase‐7. Further research showed that I‐Trp‐mediated apoptotic signalling is induced by disruption of intracellular β‐tubulin/CCT2 complexes and that I‐Trp acts as a destabiliser of microtubules.^112^ That study provided evidence showing that CCT inhibition is a potential antitumour therapeutic strategy, and suggested a novel strategy for targeting CCT: disrupting the protein‒protein interactions between CCT and its binding partners. Additionally, targeting CCT induces apoptosis through the unfolded protein response (UPR), also known as the endoplasmic reticulum (ER) stress response. In 2012, Liu et al. discovered that disrupting the β‐tubulin/CCT2 complex with I‐Trp caused ER‐associated protein and HSP90‐related protein degradation. This outcome induced ER stress‐associated apoptosis via intracellular Ca^2+^ mobilisation, mitochondrial injury and caspase overactivation.^126^ In 2017, Liu et al. demonstrated that I‐Trp selectively killed CCT2‐overexpressing cancer cells. Mechanistic studies indicated that I‐Trp induced ER stress in cancer cells through an increase in the intracellular Ca^2+^ level, activation of the UPR and MAPKs, and mitochondrial perturbation. This cascade ultimately leads to apoptosis.^127^ In 2020, a similar mechanism was found in a highly metastatic non‐small cell lung cancer (NSCLC) cell line.^128^ Although CCT2 is indispensable for I‐Trp‐induced apoptosis,^126^ a potential target of I‐Trp may lead to ER stress‐associated apoptosis, as TRiC/CCT is not commonly recognised as a stress protein. However, whether TRiC/CCT dysfunction directly leads to induction of the UPR and whether TRiC/CCT cooperates with other chaperones or proteins to induce the UPR are unknown, and further study is needed.

Another potential small molecule drug is anticarin‐β, which specifically binds to CCT4 and inhibits its expression. Both the folding and function of STAT3 are modulated by TRiC/CCT,^129^ and anticarin‐β induces apoptosis by inhibiting STAT3‐mediated antiapoptotic effects in osteosarcoma cells.^111^ In addition, CCT3 knockdown induces apoptosis in human papillary thyroid carcinoma cells.^130^ In conclusion, these studies show that the CCT subunits inhibit apoptosis in tumour cells (Figure 4B).

### 5.3 Metastasis

Epithelial–mesenchymal transition (EMT) is a plasticity‐based process related to metastasis in which mesenchymal cells acquire the phenotype of epithelial cells. After undergoing EMT, cancer cells can migrate, invade and establish distant metastases.^131^ The TGF‐β signalling pathway is dysregulated in many cancers.^132^ In a study on NSCLC, Ying et al. reported that CCT6A facilitates the TGF‐β‐induced metastasis of NSCLC cells by blocking SMAD2‐mediated suppression of metastasis. Mechanistically, CCT6A specifically interacts with the MH2 domain of SMAD2, but does not engage other R‐SMADs (such as SMADs 1, 3, 5 and 8). Furthermore, CCT6A influences the interaction between SMAD4 and SMAD2, blocking the nuclear localisation of SMAD2, and thus dampening its function as a transcriptional regulator.^82^

CCT activates the Wnt/β‐catenin signalling pathway through intracellular signalling and binding of β‐catenin and E‐cadherin. In breast cancer, knockdown of CCT2 was found to decrease the metastasis rate by preventing β‐catenin from entering the nucleus.^83^ In gastric cancer cells, CCT5 was found to promote lymph node metastasis by activating the Wnt/β‐catenin pathway through disruption of the E‐cadherin/β‐catenin adhesion complex.^133^ CCT5 bound to the cytoplasmic domain of E‐cadherin and disrupted its interaction with β‐catenin, thereby releasing β‐catenin for translocation into the nucleus.^133^

Wild‐type p53 expression is negatively related to EMT transcription factor expression and metastasis.^134^ As mentioned above, wild‐type p53 is a substrate of TRiC/CCT.^121^ The relationships between CCT subunits and wild‐type and mutated p53 are likely complex. One study indicated that CCT subunits bind to wild‐type p53 to suppress its entry into the nucleus. In CRC cells, CCT8 was found to inhibit the entry of wild‐type p53 into the nucleus and abolish its antitumour effects, promoting EMT.^113^ Interestingly, another study showed that in HCC cells, CCT1 cooperated with p53 in the cell nucleus to activate the Wnt7b/β‐catenin pathway, leading to cancer metastasis.^84^ Although CCT subunits are localised mostly in the cytoplasm, the findings of that study indicate that CCT subunits in the cell nucleus may also function as oncogenes to promote tumour formation and progression. In turn, in neck squamous cell carcinoma cells, mutated p53 was found to bind to the promoter of CCT2, forming a transcriptional network to positively regulate CCT2 expression.^135^

Hypoxia is crucial for cancer cell invasion and EMT.^136^ Gli‐1 acts as a major transcriptional activator of the hypoxia‐induced Hedgehog pathway. In CRC cells, CCT2 regulates the oncogenic protein Gli‐1 by inhibiting its ubiquitination and degradation, and promoting tumour cell invasion and migration under hypoxic conditions. Hypoxia induces the UPR, leading to an increase in the expression of HSP70. This, in turn, enhances the CCT2‐mediated folding of the Gli‐1 protein, thereby promoting cancer metastasis.^136^

**FIGURE 5:**
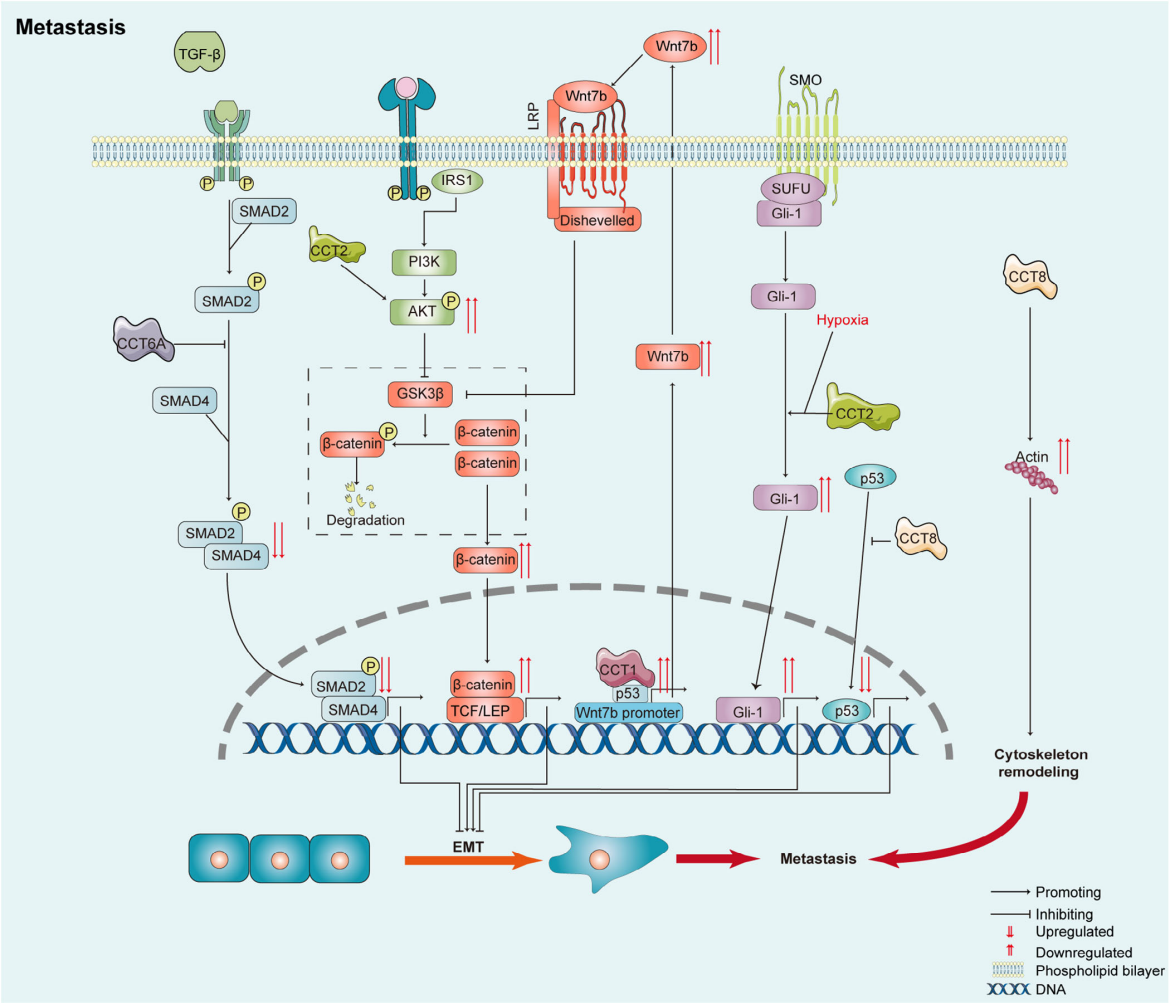
TRiC/CCT in metastasis. From left to right: In non‐small cell lung cancer cells, CCT6A suppresses metastasis through EMT by disrupting the SMAD2–SMAD4 interaction. In breast cancer cells, CCT2 promotes metastasis via the AKT–GSK3β–β‐catenin pathway. In hepatocellular carcinoma cells, CCT1 cooperates with p53 to activate the Wnt7b/β‐catenin pathway, leading to metastasis. In colorectal cancer cells, CCT2 protects Gli‐1 from degradation, promoting tumour cell invasion and migration through the hypoxia‐induced Hedgehog pathway. In colorectal cancer cells, CCT8 inhibits the nuclear entry of wild‐type p53, abolishing its antitumour effects. CCT8 increases the migration and invasion of glioma cells by controlling cytoskeletal remodelling.

The acquisition of invasive and metastatic phenotypes requires cytoskeletal remodelling.^137^, ^138^ As the cytoskeleton‐associated proteins tubulin^9^ and actin^10^ are obligate folding substrates of TRiC/CCT, some studies have indicated that CCT promotes cancer metastasis by affecting the cytoskeleton. CCT8 was found to increase the migration and invasion rates of glioma cells by controlling the reorganisation of the cytoskeleton.^139^ In human oesophageal squamous cell carcinoma cells, CCT8 was found to positively regulate α‐actin and β‐tubulin levels to promote cell migration and invasion.^140^ These findings indicate that targeting CCT may affect the cytoskeleton and has potential therapeutic effects on metastatic tumour cell dissemination. Interestingly, I‐Trp was shown to disrupt the protein‒protein interaction between β‐tubulin and CCT2 to suppress the migration and invasion of lung adenocarcinoma cells.^128^ Knockdown of CCT2 was found to decrease the metastasis rate of breast cancer cells by inhibiting the activity and expression of MMP2/9, which act as matrix metalloproteases to degrade the extracellular matrix.^83^ In conclusion, the CCT subunits promote metastasis by promoting EMT and controlling cytoskeletal remodelling (Figure 5).

### 5.4 Drug resistance

**FIGURE 6:**
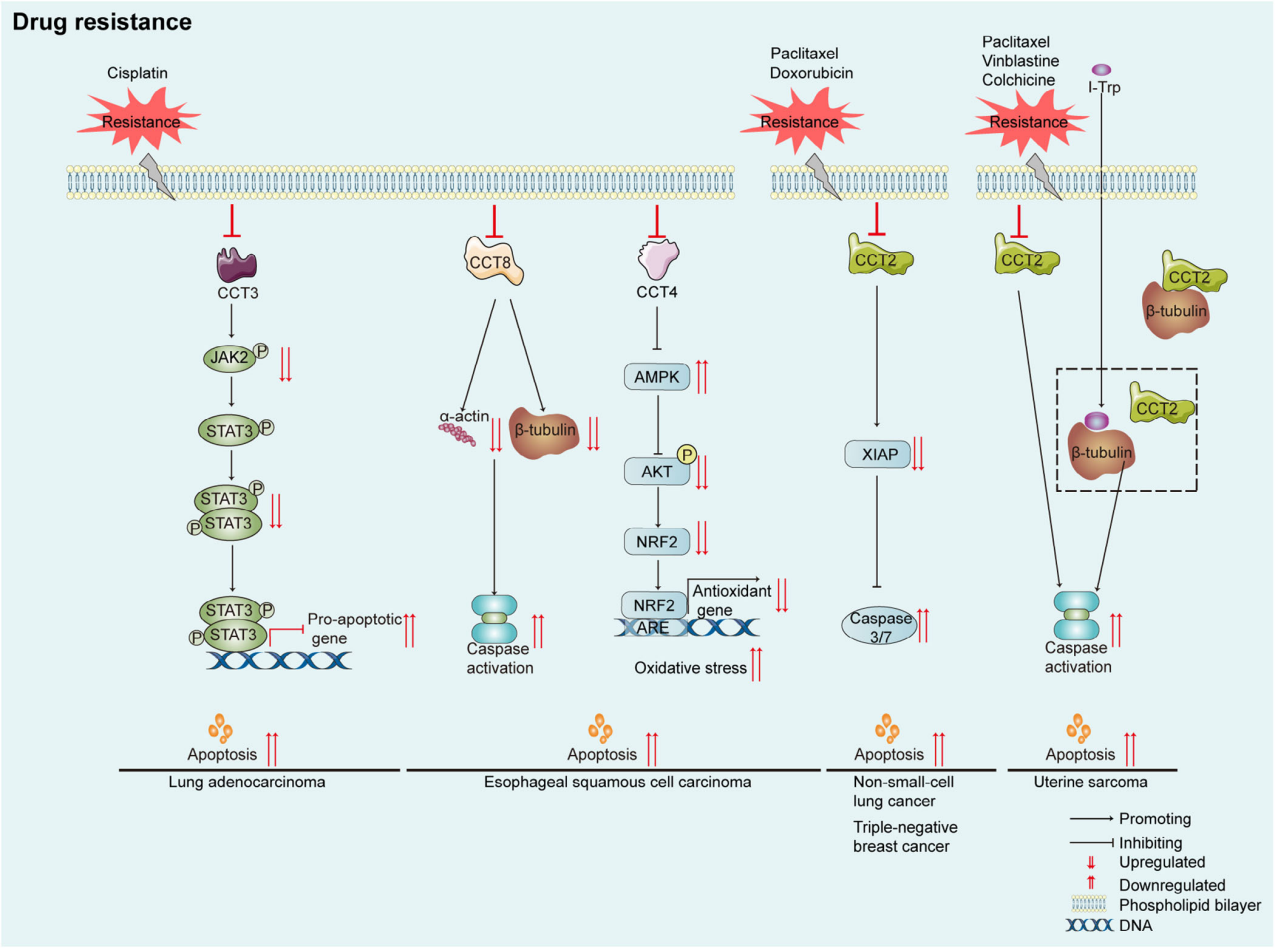
TRiC/CCT in drug resistance. From left to right: In lung adenocarcinoma, CCT3 knockdown resensitises cells to cisplatin via the JAK2/STAT3 pathway. In oesophageal squamous cell carcinoma, CCT8 knockdown resensitises cells to cisplatin by regulating α‐actin and β‐tubulin, thereby inducing apoptosis. Moreover, CCT4 knockdown resensitises cells to cisplatin via the AMPK/AKT/NRF2 pathway, leading to increased intracellular oxidative stress, and ultimately inducing apoptosis. CCT2 knockdown in triple‐negative breast cancer and non‐small cell lung cancer cells increases doxorubicin and paclitaxel sensitivity, inducing caspase activation through XIAP. CCT2 knockdown sensitises multidrug‐resistant uterine sarcoma cells to paclitaxel, vinblastine and colchicine. Additionally, I‐Trp disrupts the binding of CCT2 to β‐tubulin, further contributing to sensitivity to paclitaxel, vinblastine and colchicine.

Drug resistance is a major cause of chemotherapy failure and increased cancer‐related mortality. According to recent findings, combining CCT subunit inhibitors with other therapies, such as microtubule‐targeting agents and cisplatin, enhances the curative effect of these treatments in patients with cancer. Since 1984, taxanes have been extensively used as microtubule‐targeting antitumour agents. However, the antitumour efficacy of these drugs has historically been limited by chemoresistance.^141^ Tubulin is an obligate folding substrate of TRiC/CCT,^9^ and targeting the β‐tubulin/CCT2 interaction promotes cancer cell apoptosis^112^; thus, the combination of CCT and microtubule‐targeting agents (including doxorubicin, paclitaxel, vincristine, vinblastine and colchicine) can overcome chemoresistance to achieve clinical success. Silencing of CCT2 expression in the MDA‐MB‐231 triple‐negative breast cancer cell line and the highly metastatic CL1‐5 NSCLC cell line sensitised them to doxorubicin and paclitaxel. Moreover, CCT2 directly binds to and stabilises XIAP and β‐catenin, thereby increasing their levels and causing chemoresistance.^83^ CCT3 knockdown increases the sensitivity of HCC cells to chemotherapeutic drugs such as the microtubule‐destabilising drug vincristine while reducing their sensitivity to the microtubule‐stabilising drug paclitaxel.^119^ Interestingly, although the β‐tubulin/CCT2 complex was constitutively formed, its assembly was disrupted after exposure to I‐Trp. In addition, multidrug‐resistant MES‐SA/Dx5 uterine sarcoma cells acquired resistance to the β‐tubulin‐binding agents paclitaxel, vinblastine and colchicine.^142^ Knockdown of CCT2 desensitised these multidrug‐resistant cells to I‐Trp, which caused apoptotic cell death.^112^ In oesophageal squamous cell carcinoma cells, CCT8 knockdown significantly increased the apoptosis rate after cisplatin treatment. CCT8 overexpression was found to be related to poor prognosis and cisplatin resistance.^140^ Knockdown of CCT4 increased the sensitivity of oesophageal squamous cell carcinoma cells to cisplatin by regulating the AMPK/AKT/NRF2 signalling pathway.^114^ In lung adenocarcinoma, CCT3 knockdown resensitised cancer cells to cisplatin by inhibiting the Janus kinase 2 (JAK2)/STAT3 pathway^143^ (Figure 6).

### 5.5 Cancer exosomes

Exosomes, which can deliver drug payloads into cells, are extracellular membrane vesicles secreted by almost all cell types, including cancer cells, into almost all bodily fluids. They consist of a spherical proteolipid bilayer membrane, and contain specific proteins, RNA molecules and DNA molecules.^144^ Recent studies have demonstrated that CCT4 is a key regulator of vesicle trafficking,^145^ and the levels of CCT subunits were found to be upregulated in various cancer exosomes.^146^, ^147^ Additionally, the levels of six of the eight CCT subunits (CCT1, CCT2, CCT3, CCT5, CCT6A and CCT7) were found to be significantly increased in glioblastoma cell‐derived extracellular vesicles, and CCT6A in glioblastoma tissue showed a potential link with EGFR and was identified as a potential prognostic biomarker.^146^ Similarly, CCT8 was found to be overexpressed in HCC cell‐derived exosomes. The combination of three serum markers (CCT8, CFL1 and AFP) demonstrated a diagnostic advantage in HCC.^147^ Although an increasing number of CCT subunits have been found in various cancer exosomes, their exact roles and mechanisms of action in cancer exosomes are still unknown.

### 5.6 Metabolic reprogramming

**FIGURE 7:**
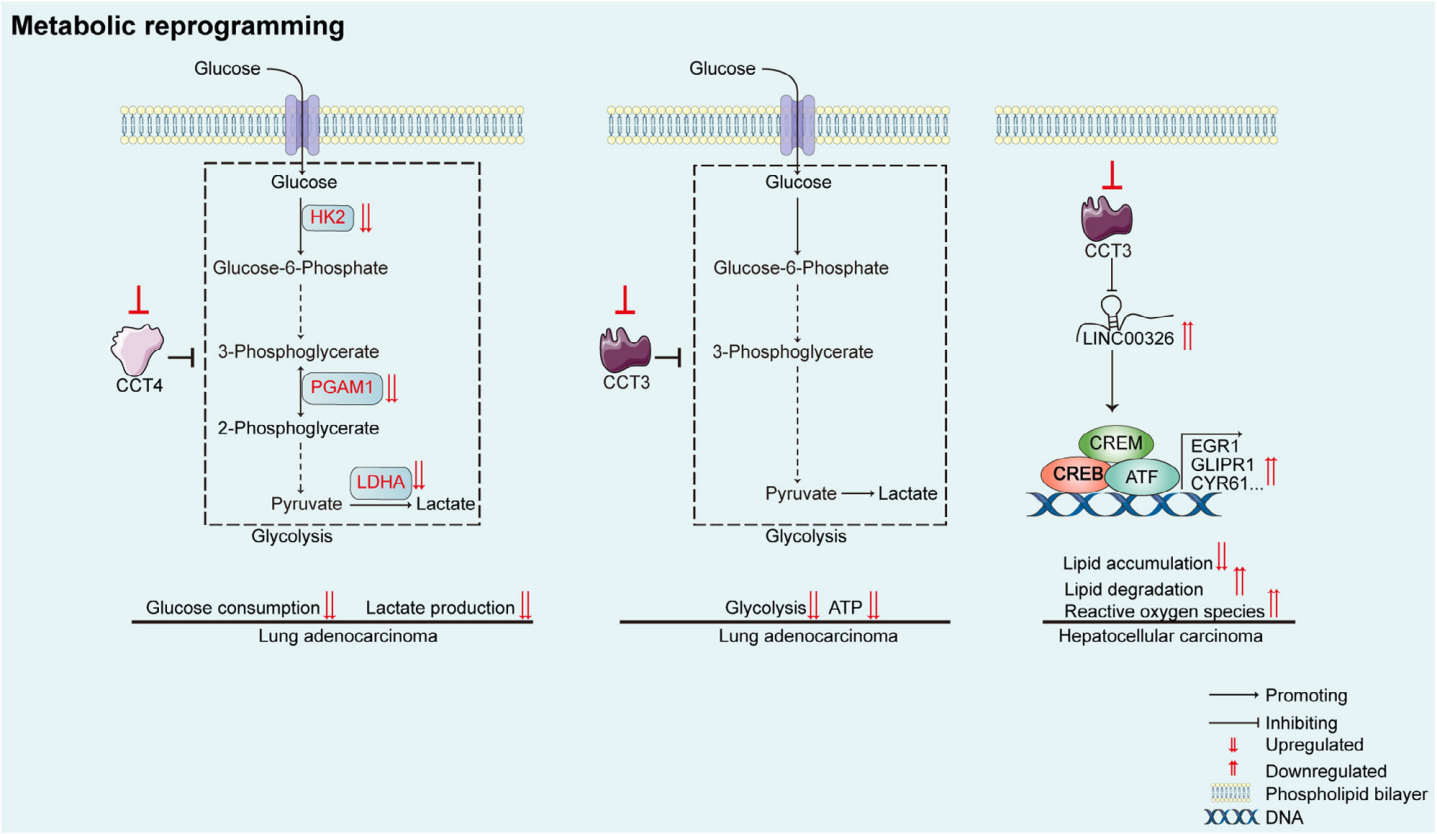
Effect of TRiC/CCT on metabolic reprogramming. In oesophageal squamous cell carcinoma, CCT4 knockdown reduces glycolytic activity by regulating key proteins associated with glucose metabolism, including hexokinase‐2 (HK2), phosphoglycerate mutase 1 (PGAM1) and lactate dehydrogenase A (LDHA). In lung adenocarcinoma, CCT3 knockdown reduces tumour growth by decreasing the level of intracellular ATP generated through glycolysis. In hepatocellular carcinoma, targeting the CCT3–LINC00326 network inhibits tumour growth by regulating lipid metabolism (reducing lipid accumulation, enhancing lipid degradation and increasing reactive oxygen species generation). The CCT3–LINC00326 axis transcriptionally regulates genes associated with lipid metabolism, including EGR1, CYR61 and GLIPR1, through the activity of transcription factors such as CREM, CREB and ATF. ATF, activating transcription factor; CREM, cAMP response element modulator; CREB, cAMP response element‐binding protein; CYR61, cysteine‐rich angiogenic inducer 61; EGR1, early growth response protein 1; GLIPR1, glioma pathogenesis‐related protein 1.

Metabolic reprogramming allows tumour cells to meet the increased energy demands for rapid proliferation, invasion and metastasis.^148^ CCT subunits in tumour cells can regulate the reprogramming of glucose and lipid metabolism at the transcriptional and signalling levels. In HCC, CCT3 accelerates tumour growth by regulating lipid metabolism.^149^ The CCT3–LINC00326 axis increases lipid accumulation, decreases lipid degradation and inhibits the generation of ROS.^149^ Interestingly, CCT3 expression was found to be upregulated in patients with lipid metabolism disorders (metabolic‐associated fatty liver disease and nonalcoholic steatohepatitis), as determined via analysis of publicly available data.^149^ However, the role of CCT3 in metabolic‐associated fatty liver diseases has not been fully elucidated and deserves further study. In lung adenocarcinoma, knockdown of CCT3 was found to inhibit tumour growth and metastasis by decreasing the amount of intracellular ATP produced via glycolysis.^150^ Moreover, knockdown of CCT4 increased the sensitivity of oesophageal squamous cell carcinoma cells to cisplatin by targeting glucose metabolism and regulating the expression of key proteins in the glycolytic pathway, such as hexokinase‐2 (HK2), lactate dehydrogenase A (LDHA) and phosphoglycerate mutase 1 (PGAM1)^114^ (Figure 7).

## 6 CONCLUSIONS

Since the discovery of TRiC/CCT more than four decades ago,^151^ significant progress has been made in understanding the structure of TRiC/CCT, its role in mediating protein folding, and its implications for health and disease. Recent studies have revealed an increasing number of roles played by TRiC/CCT in multiple biological activities (including cell cycle regulation, initiation of transcription and translation, cellular immortality, epigenetics, adaptive immunity, autophagy, and signal transduction). Additionally, it is involved in the pathogenesis of many diseases, such as neuropathies, cardiovascular diseases, viral and bacterial infections, and cancers. Targeting TRiC/CCT has also shown promising diagnostic and therapeutic potential in these diseases.

However, questions remain regarding the substrate selectivity and folding mechanism of TRiC/CCT and the mechanism through which TRiC/CCT cooperates with other components of the protein homeostasis network to maintain proteostasis. Moreover, although TRiC/CCT shows diagnostic and therapeutic potential, the ability of the related findings to be translated from the laboratory to clinical settings requires further validation through clinical cohort studies and prospective trials. Additionally, future studies should focus on designing new analytical tools and utilising TRiC/CCT as a biomarker not only for diagnosing disease, but also for monitoring therapeutic responses and disease status. Recently, TRiC/CCT has been shown to be involved in cancer exosomes, drug resistance, cancer metabolism and T‐cell immunity, and to establish a potential connection between cancer cells and the tumour microenvironment (T‐cell exhaustion^152^ and cancer cell resistance to IFNγ^153^). However, the relationship between TRiC/CCT and the tumour microenvironment (which involves various cell types, such as natural killer cells, monocytes, myeloid cells, B cells and T cells) has not been fully elucidated and merits further investigation. In the contexts of tumour metabolism and tumour‐derived exosomes, the potential roles of TRiC/CCT have only just begun to be revealed, and deserve further in‐depth study. Finally, considering the involvement of TRiC/CCT in tumour drug resistance, determining whether TRiC/CCT is involved in immune checkpoint inhibitor resistance is worthy of exploration.

## AUTHOR CONTRIBUTIONS

Binhao Zhang and Yonglong Pan initiated the study. Chenglong Zeng, Yonglong Pan and Binhao Zhang drafted the manuscript. Chenglong Zeng, Zhao Huang and Shenqi Han prepared the figures. Bixiang Zhang and Zhao Huang revised the manuscript. Bixiang Zhang, Zhao Huang and Yonglong Pan obtained funding. All the authors read and approved the final manuscript.

## CONFLICT OF INTEREST STATEMENT

The authors declare that they have no conflicts of interest.

## ETHICS STATEMENT

Not applicable.
